# Associations between composite dietary antioxidant index and estimated 10-year atherosclerotic cardiovascular disease risk among U.S. adults

**DOI:** 10.3389/fnut.2023.1214875

**Published:** 2023-08-10

**Authors:** Jia Zhang, Xueqin Lu, Ruifeng Wu, Hanchen Ni, Lingli Xu, Wenjuan Wu, Cheng Lu, Jiayi Feng, Yongmei Jin

**Affiliations:** ^1^Department of Cardiovascular Medicine, Shanghai Seventh People’s Hospital, Shanghai University of Traditional Chinese Medicine, Shanghai, China; ^2^Department of Nutrition, Shanghai Seventh People’s Hospital, Shanghai University of Traditional Chinese Medicine, Shanghai, China; ^3^Department of Pediatrics, Shanghai Seventh People’s Hospital, Shanghai University of Traditional Chinese Medicine, Shanghai, China; ^4^Department of Nursing, Shanghai Seventh People’s Hospital, Shanghai University of Traditional Chinese Medicine, Shanghai, China; ^5^Intensive Care Unit, Shanghai Seventh People’s Hospital, Shanghai University of Traditional Chinese Medicine, Shanghai, China; ^6^Department of Gastrointestinal Diagnosis and Treatment, Shanghai Seventh People’s Hospital, Shanghai University of Traditional Chinese Medicine, Shanghai, China

**Keywords:** composite dietary antioxidant index, atherosclerotic cardiovascular disease, National Health and Nutrition Examination Survey, American, adults (MeSH)

## Abstract

**Background:**

Atherosclerotic cardiovascular disease (ASCVD) remains the leading cause of death and disability both in U.S. and worldwide. Antioxidants have been proved critical in mitigating the development of atherosclerosis. This study aimed to investigate the associations between composite dietary antioxidant index (CDAI) and estimated 10-year ASCVD risk among U.S. adults.

**Methods:**

Data extracted from the National Health and Nutrition Examination Survey were analyzed. A total of 10,984 adults aged 18 years and above were included in this study. CDAI was calculated based on the dietary intake reported in their 24-h recall interviews. The estimated 10-year ASCVD risk was calculated via Pooled Cohort Equations (PCE).

**Results:**

After adjusting potential confounders, it was indicated that CDAI score was negatively correlated with 10-year ASCVD risk (OR 0.97, 95% CI 0.95–0.99). Stratify CDAI score by quartile, results showed that participants in the second, third, and fourth quartiles had lower ASCVD odds ratio (Q2: OR 0.87, 95% CI 0.69–1.09; Q3: OR 0.78, 95% CI 0.62–0.98; Q4: OR 0.74, 95% CI 0.59–0.94) than those in the first quartile (Q1, lowest CDAI score group), which was confirmed by the trend test as well (*p* < 0.05). Subgroup analyses stratified by sex, age, race/ethnicity, and smoking status did not show significant effect modification.

**Conclusion:**

Higher dietary antioxidants intake is associated with lower ASCVD risk among U.S. adults, for which policymakers and healthcare professionals may consider increasing the consumption of antioxidant-rich foods as a preventive strategy for ASCVD.

## 1. Introduction

Atherosclerotic cardiovascular diseases (ASCVD), which involve stroke, myocardial infarction and sudden cardiac death (1), take up a large proportion of healthcare budgets and is a significant financial burden worldwide (2). In the United States, it is also the leading cause of death with an estimated medical cost over $200 billion annually. The reason for this is mainly because prevention strategies are not being implemented effectively, and a significant number of adults have uncontrolled risk factors for ASCVD.2 Therefore, identifying high-risk ASCVD population is significant for its primary prevention. 2013 American College of Cardiology/American Heart Association (ACC/AHA) Guideline proposed Pooled Cohort Equations (PCE) to estimate the 10-year risk of developing a first ASCVD event, which was widely recommended by amount of guidelines as a reliable tool for ASCVD’s 10-year risk assessment. PCE’s initial risk scoring for ASCVD was also of vital importance. In addition, PCE was also recommended by hypertension guideline (3) to instruct pharmacotherapy usage.

High oxidative stress conditions can cause multiple oxidative modifications to lipoprotein phospholipids, which are closely linked to the onset and progression of ASCVD (4). Thus maintaining oxidative homeostasis and an antioxidant defense system is essential for ASCVD prevention. Diet is known to be a major risk factor for most cardiovascular diseases, and an adequate intake of antioxidants could help reduce oxidative burden (5). The composite dietary antioxidant index (CDAI) comprises vitamins A, C, and E, zinc, selenium, and carotenoids as dietary antioxidants. It is a comprehensive score reflecting an individual’s antioxidant profile.6 CDAI was proposed by Wright et al. in 20,047 to evaluate the overall impact of antioxidants on health.

The effect of total dietary antioxidant capacity on health has attracted more and more attention in recent years because of antioxidant’s critical role in global diet patterns (6–9). However, only a limited number of studies have investigated the association between CDAI and cardiovascular diseases. These studies focused either on CDAI and all-cause and cardiovascular mortality or on the association between high dietary antioxidant intake (one or more nutrients such as vitamins A, C, and D, zinc, and carotenoid) and ASCVD risk (10–12). The effect of CDAI on estimated 10-year ASCVD risk remains unclear. This study aimed to explore the association between CDAI and estimated 10-year ASCVD risk based on a national sample of U.S. adults.

## 2. Materials and methods

### 2.1. Study population

**Figure 1:**
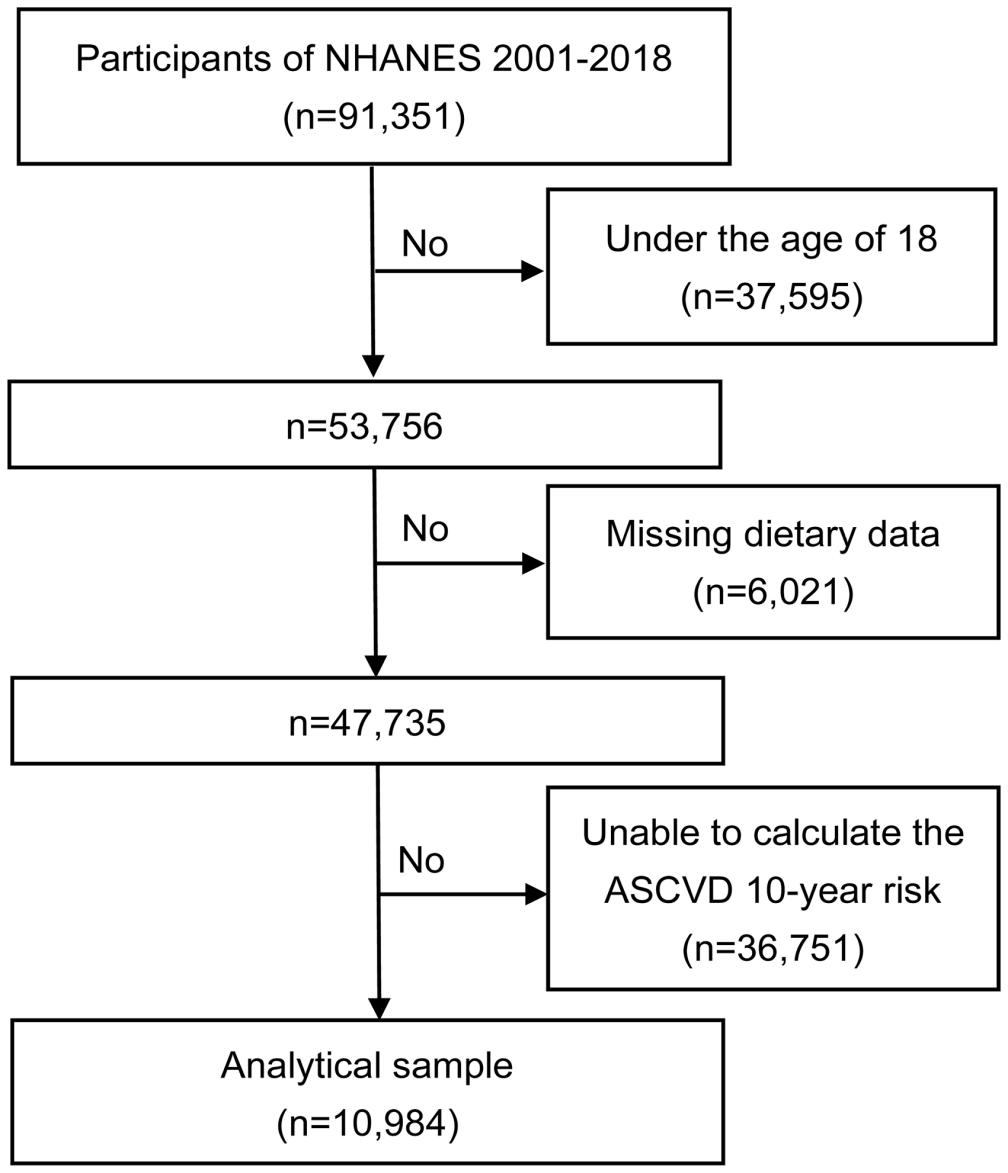
Flowchart of the study. ASCVD, atherosclerotic cardiovascular disease.

The National Health and Nutrition Examination Survey (NHANES) is a large cross-sectional survey interviewing a group of representative non-institutionalized U.S. civilians. This survey employed a stratified complex multi-stage probability survey design and requested its participants to provide detailed information about their dietary intake for two consecutive 24-h periods. The first dietary recall was completed at a mobile examination center while the second one was accomplished by phone in 3–10 days. To minimize error, all interviewers were intensively trained for one week (10). To ensure the consistency in measurement of food consumption, respondents were provided with a standard set of measuring tools such as cups, spoons, glasses, and bottles. The U.S. Department of Agriculture (USDA) Automated Multiple-Pass Method was utilized to gather two 24-h dietary recalls. The Research Ethics Review Committee of the National Center for Health Statistics has approved the NHANES study, and all participants had been given informed written consent. Data of NHANES 2001–2018 cycle were selected for analysis. In this study, a total of 91,351 participants were selected. Exclusion criteria included: individuals under the age of 18 years old (*n* = 37,595); individuals with missing dietary data (*n* = 6,021); individuals whose data were insufficient for ASCVD 10-year risk calculation (*n* = 36,751). Data of 10,984 patients were ultimately included in this analysis (Figure 1).

### 2.2. Calculation of CDAI

24-h dietary recall interviews were used to collect information on intake of dietary antioxidant and other food components. During the interview, participants were asked to recall specific food and drinks they consumed within the 24-h period before the interview. To evaluate the overall exposure to dietary antioxidants, a modified version of Composite Dietary Antioxidant Index (CDAI) developed by Wright et al. was used (13, 14). The standardized intake of six antioxidant nutrients (vitamin A, vitamin C, vitamin E, zinc, selenium, and carotenoids) was calculated by dividing the difference between individual intake and mean by the standard deviation, and their sum was used to represent CDAI. The formula was shown as following:

$\mathrm{CDAI}=\overset{6}{\underset{i=1}{\sum }}\frac{\mathrm{Individual}\;\mathrm{Intake}-\mathrm{Mean}}{\mathrm{SD}}$

It should be noted that the dietary antioxidant intake did not include those from supplements, medications, or plain drinking water.

### 2.3. Assessment of ASCVD risk

Pooled Cohorts Equations model from 2013 ACC/AHA guidelines was applied to assess the 10-year risk of ASCVD.1 This model takes demographics, blood cholesterol, blood pressure, smoking, and diabetes history into consideration to predict the likelihood of a first-time hard ASCVD event. Incorporating several recommendations, 7.5% was taken as the cutoff value for 10-year ASCVD risk in this study.

### 2.4. Covariates

Covariates were adopted to reduce deviation, which included demographic information such as age, sex, educational level, and poverty income ratio (PIR), lifestyle information such as smoking and drinking habits, physical examination results such as body mass index (BMI), and self-reported health status such as medical and drug history. NHANES calculated BMI based on height and weight measurements. PIR was calculated by dividing the family income by the poverty threshold, and results were categorized into three levels: low income (< 1.3), moderate income (1.3–3.5), and high income (> 3.5). Smoking status was divided into never (less than 100 cigarettes in lifetime), former (more than 100 cigarettes in lifetime and had quit smoking at the time of the survey), and current (more than 100 cigarettes in lifetime and was still smoking every several days at least). Current drinking status was classified into heavy drinking (≥ 3 drinks per day for females; ≥ 4 drinks per day for males; binge drinking for 5 or more days per month), moderate drinking (≥ 2 drinks per day for females; ≥ 3 drinks per day for males; binge drinking ≥2 days per month) (15), and mild drinking (other than the above two). Hypertension was characterized by a blood pressure reading of ≥140/90 mmHg, a medical diagnosis of hypertension, or self-reported use of antihypertensive medication in health questionnaires. Diabetes was confirmed if patients met one or more of the following criteria: (1) diagnosis of diabetes reported by their doctors, (2) glycohemoglobin (HbA1c) > 6.5%, (3) fasting blood glucose ≥7.0 mmoL/L, (4) random blood glucose ≥11.1 mmoL/L, or (5) two-hour blood glucose ≥11.1 mmol/L in oral glucose tolerance test (OGTT). Chronic kidney disease (CKD) was confirmed according to KDIGO guideline (16). We calculated the estimated glomerular filtration rate (eGFR) using the serum creatinine equation from the Chronic Kidney Disease Epidemiology Collaboration (CKD-EPI) study (17).

### 2.5. Statistical analysis

Participants were categorized into two groups based on their 10-year ASCVD risks: one group with a risk of less than 7.5% and the other group with 7.5% or higher. Baseline data differences between these two groups were compared. Continuous variables were expressed as means ± standard errors, and categorical variables were represented by percentages. Weighted linear regression was used for continuous variables while weighted chi-squared tests for categorical variables. The relationship between CDAI and 10-year ASCVD risk was investigated by multivariable logistic regression equations. The consistency of the relationship was tested via linear trend tests. Generalized additive models (GAMs) and smooth curve fittings were used to explore non-linear association. Subgroup analyses and interactions were conducted for covariates such as age, sex, hypertension, diabetes, and BMI with controlled variables. All statistical analyses were conducted using R (version 3.5.3) and EmpowerStats,^1^ and *p* < 0.05 was taken as statistically significant.

## 3. Results

### 3.1. Baseline characteristics

The baseline characteristics were presented in Table 1, which revealed certain difference between two groups. More specifically, participants of “10-year ASCVD risk ≥7.5%” group tended to be older, female, and have higher BMI, worse economic status, lower educational level, smoking and drinking history, diabetes and hypertension, antidiabetic and antihypertensive medications, and lower CDAI.

**Table 1:** Baseline characteristics of subjects.

Characteristics	Total *n* = 10,984	10-year risk of ASCVD < 7.5% *n* = 5,988	10-year risk of ASCVD ≥ 7.5% *n* = 4,996	*p*-value
Age (years)	58.85 ± 11.37	51.91 ± 8.23	67.17 ± 8.74	<0.05
Sex, *n* (%)				<0.05
Male	5,581 (50.81%)	3,825 (63.88%)	1756 (35.15%)	
Female	5,403 (49.19%)	2,163 (36.12%)	3,240 (64.85%)	
BMI (kg/m^2^)	29.35 ± 6.60	28.93 ± 6.47	29.86 ± 6.73	<0.05
PIR^a^, *n* (%)				<0.05
Low	2,296 (22.11%)	1,106 (19.43%)	1,190 (25.37%)	
Medium	3,568 (34.36%)	1,574 (27.65%)	1994 (42.52%)	
High	4,519 (43.52%)	3,013 (52.92%)	1,506 (32.11%)	
Education level, *n* (%)				<0.05
Less than high school	1711 (15.58%)	690 (11.53%)	1,021 (20.44%)	
High school	2,899 (26.40%)	1,406 (23.49%)	1,493 (29.90%)	
More than high school	6,369 (58.01%)	3,889 (64.98%)	2,480 (49.66%)	
Smoking, *n* (%)				<0.05
Never	4,856 (44.21%)	2,867 (47.88%)	1989 (39.81%)	
Former	3,815 (34.73%)	1951 (32.58%)	1864 (37.31%)	
Now	2,313 (21.06%)	1,170 (19.54%)	1,143 (22.88%)	
Drinking^b^, *n* (%)				<0.05
Never	985 (9.43%)	350 (6.12%)	635 (13.44%)	
Former	2,165 (20.73%)	932 (16.29%)	1,233 (26.10%)	
Mild	4,313 (41.29%)	2,411 (42.14%)	1902 (40.26%)	
Moderate	1,559 (14.92%)	984 (17.20%)	575 (12.17%)	
Heavy	1,424 (13.63%)	1,045 (18.26%)	379 (8.02%)	
Diabetes, *n* (%)	2056 (18.72%)	502 (8.38%)	1,554 (31.10%)	<0.05
Hypertension, n (%)	5,676 (51.68%)	2,237 (37.36%)	3,439 (68.84%)	<0.05
CKD, *n* (%)	2,179 (20.00%)	577 (9.69%)	1,602 (32.42%)	<0.05
Antidiabetic medications, *n* (%)	1,284 (11.69%)	317 (5.29%)	967 (19.36%)	<0.05
Antihypertensive medications (*n*, %)	1,307 (11.90%)	397 (6.63%)	910 (18.21%)	<0.05
CDAI	0.57 ± 3.98	0.94 ± 4.20	0.12 ± 3.66	<0.05

^a^PIR was calculated by dividing the income of the family by the poverty threshold of the family and was categorized on three levels: <1.3 (low income), 1.3–3.5 (moderate income), and > 3.5 (high income); ^b^Current drinking is subdivided into three categories: heavy drinking (≥3 drinks per day for females; ≥4 drinks per day for males; binge drinking on 5 or more days per month), moderate drinking (≥2 drinks per day for females; ≥3 drinks per day for males; binge drinking ≥ 2 days per month) and mild drinking (not meet the above). ASCVD, atherosclerotic cardiovascular disease; BMI, body mass index; PIR, poverty income ratio; CKD, chronic kidney disease; CDAI, composite dietary antioxidant index.

To investigate the dietary factors causing the variation of CDAI between two groups, we displayed every single component score of CDAI in Table 2. Individuals with a 10-year ASCVD risk ≥7.5% had lower antioxidant dietary scores than the other group regarding all components of CDAI.

**Table 2:** Comparison of each component of CDAI scores between individuals with 10-year risk of ASCVD <7.5% and individuals with 10-year risk of ASCVD ≥7.5%.

Characteristics	Total	10-year risk of ASCVD < 7.5%	10-year risk of ASCVD ≥ 7.5%	*p*-value
Vitamin A	474.95 (467.14, 482.90)	483.51 (472.51, 494.76)	464.92 (453.97, 476.13)	<0.05
Vitamin C	44.12 (43.09, 45.17)	44.95 (43.54, 46.40)	43.14 (41.66, 44.68)	0.09
Vitamin E	6.46 (6.37, 6.54)	7.07 (6.95, 7.20)	5.79 (5.68, 5.90)	<0.05
Zinc	9.95 (9.84, 10.06)	10.87 (10.71, 11.04)	8.94 (8.80, 9.08)	<0.05
Selenium	93.42 (92.42, 94.44)	102.87 (101.35, 104.42)	83.24 (82.00, 84.49)	<0.05
Carotenoid	4401.68 (4284.10, 4522.48)	4735.84 (4565.78, 4912.24)	4032.39 (3873.73, 4197.55)	<0.05

Data are presented as the geometric mean and 95% confidence interval. CDAI, composite dietary antioxidant index; ASCVD, atherosclerotic cardiovascular disease.

### 3.2. Association between CDAI score and 10-year ASCVD risk

**Figure 2:**
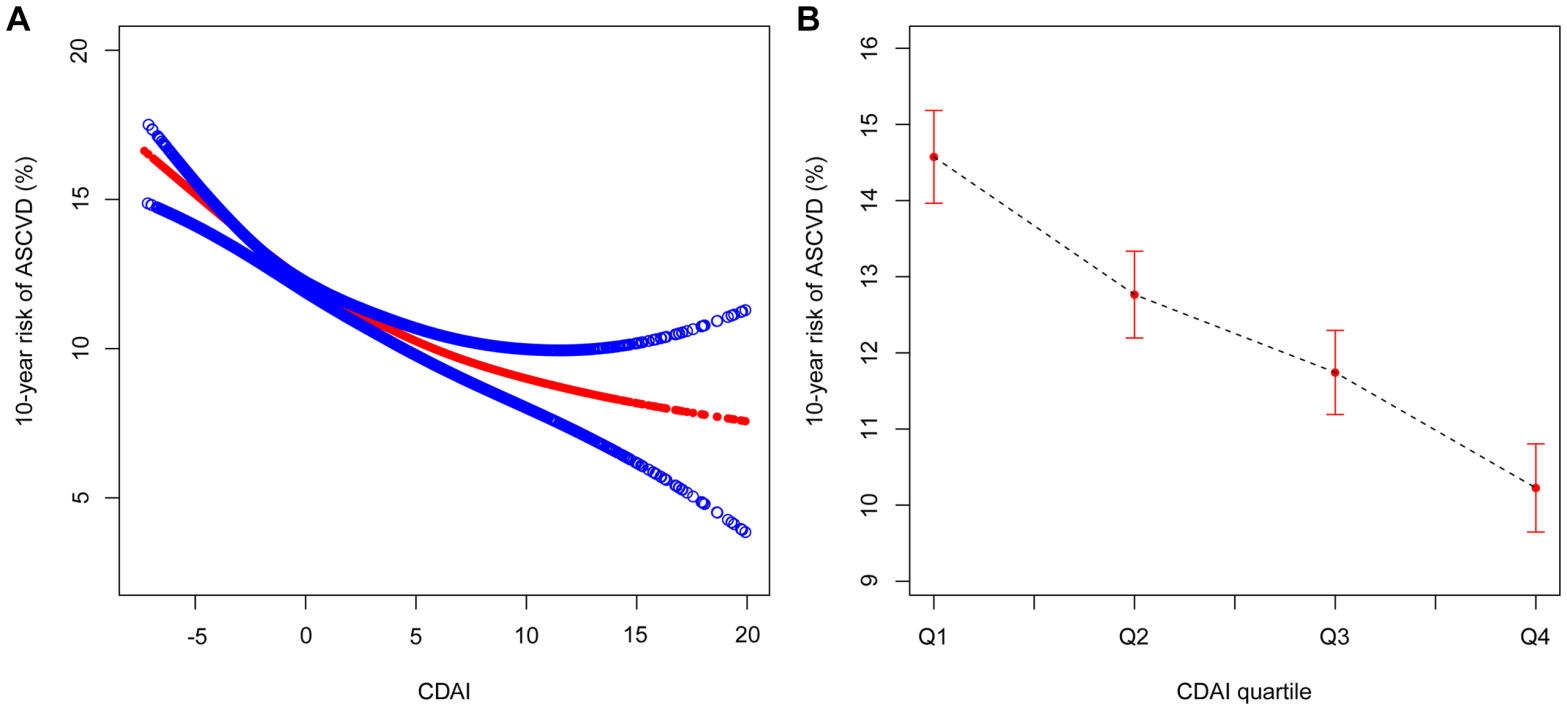
The association between CDAI [**(A)** as continuous variable; **(B)** as categorical variable] and 10-year risk of ASCVD. The red line represents the best-fit line, and the blue lines are 95% CI. CDAI, composite dietary antioxidant index; ASCVD, atherosclerotic cardiovascular disease; CI, confidence interval.

The logistic regression modeling results displayed in Table 3 demonstrated the correlation between CDAI score and 10-year ASCVD risk. After adjusting for covariates (age, sex, PIR, educational level, BMI, smoking, drinking, diabetes, hypertension, CKD, antidiabetic medications and antihypertensive medications), it was indicated that CDAI score was negatively correlated with 10-year ASCVD risk (OR 0.97, 95% CI 0.95–0.99). After stratifying score by quartile, results showed that participants in the second, third, and fourth quartiles had lower ASCVD odds ratio (Q2: OR 0.87, 95% CI 0.69–1.09; Q3: OR 0.78, 95% CI 0.62–0.98; Q4: OR 0.74, 95% CI 0.59–0.94) than those in the first quartile (Q1, lowest CDAI score group), which was confirmed by the trend test as well (*p* < 0.05). We also used generalized additive models and smooth curve fittings to evaluate the associations between these two items. When CDAI was treated as a continuous variable, a negative correlation was observed between CDAI and 10-year ASCVD risk (Figure 2A). When CDAI was treated as a categorical variable with four quartiles, the relationship between CDAI and 10-year ASCVD risk remained unchanged (Figure 2B).

**Table 3:** Odd ratios and 95% confidence intervals for 10-year risk of ASCVD according to CDAI.

Characteristics	Model 1	Model 2	Model 3
Continuous	0.94 (0.94, 0.95)	0.95 (0.94, 0.97)	0.97 (0.95, 0.99)
Quartile			
Q1	Reference	Reference	Reference
Q2	0.83 (0.74, 0.92)	0.86 (0.72, 1.03)	0.87 (0.69, 1.09)
Q3	0.67 (0.61, 0.75)	0.67 (0.56, 0.80)	0.78 (0.62, 0.98)
Q4	0.55 (0.49, 0.61)	0.61 (0.51, 0.73)	0.74 (0.59, 0.94)
*p* for trend	<0.05	<0.05	<0.05

Model 1: Non-adjusted. Model 2: Adjusted for age, sex, and PIR. Model 3: Adjusted for age, sex, PIR, education level, BMI, smoking, drinking, diabetes, hypertension, CKD, antidiabetic medications, and antihypertensive medications. ASCVD, atherosclerotic cardiovascular disease; CDAI, composite dietary antioxidant index; PIR, poverty income ratio; BMI, body mass index; CKD, chronic kidney disease.

**Figure 3:**
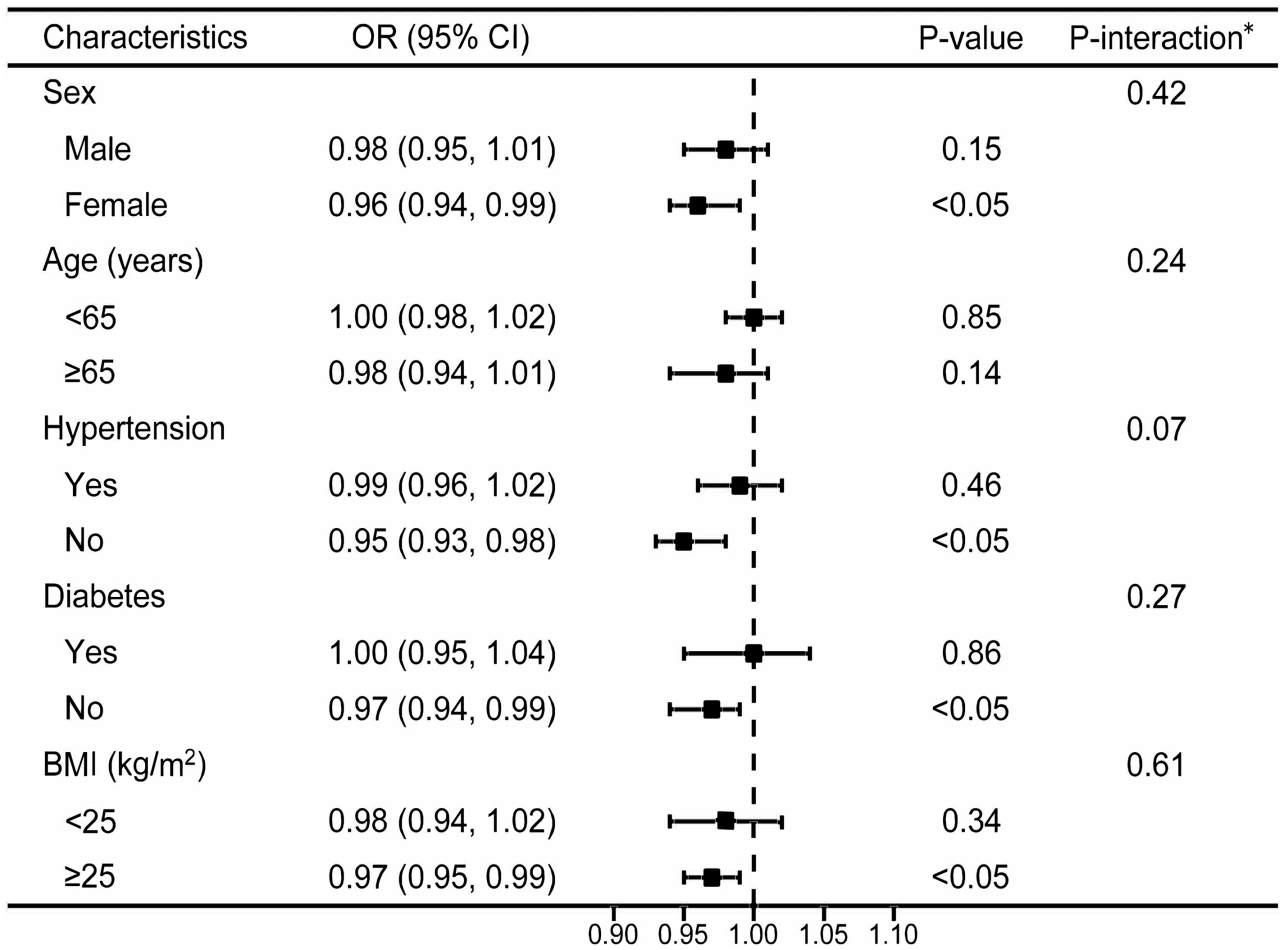
Stratified analyses between CDAI and 10-year risk of ASCVD using logistic regression. ^*^Each stratification adjusted for all the factors (age, sex, PIR, education level, BMI, smoking, drinking, diabetes, hypertension, CKD, antidiabetic medications and antihypertensive medications) except the stratification factor itself. OR, odd ratio; CI, confidence interval; ASCVD, atherosclerotic cardiovascular disease; CDAI, composite dietary antioxidant index; PIR, poverty income ratio; BMI, body mass index; CKD, chronic kidney disease.

**Figure 4:**
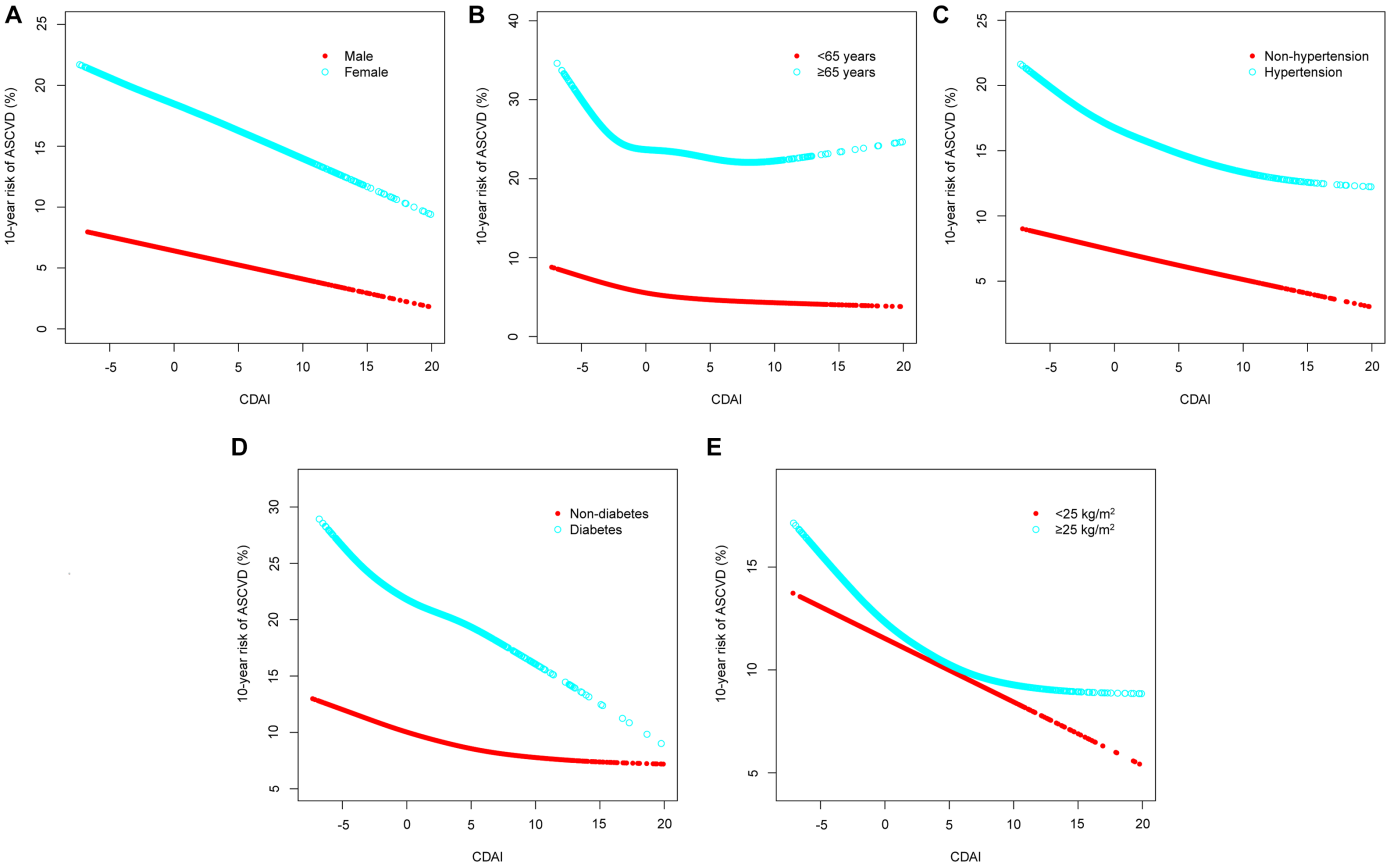
Stratified analyses [by **(A)** sex; **(B)** age; **(C)** hypertension; **(D)** diabetes; **(E)** BMI] between CDAI and 10-year risk of ASCVD using generalized additive model and smooth curve fittings. BMI, body mass index; ASCVD, atherosclerotic cardiovascular disease; CDAI, composite dietary antioxidant index.

The forest plot showed that all stratification variables had consistent interactions, i.e., a negative correlation between CDAI score and 10-year ASCVD risk existing in all stratified analyses (Figure 3). As a sensitivity analysis, the relationship between CDAI score and 10-year risk of ASCVD was also examined in stratified analysis of smooth curve fittings, which suggested a negative correlation between the two variables regardless of gender, age (above or below 65 years), hypertension status, diabetes status, and BMI (above or below 25 kg/m^2^) (Figure 4).

## 4. Discussion

It was demonstrated that overall antioxidant intake, measured by CDAI, was in significant negative association with ASCVD after adjusting for multiple covariates. Participants in the highest CDAI quartile showed a reduced 10-year ASCVD risk than those in the lowest quartile. This study is the first one analyzing the association between CDAI and PCE estimated 10-year ASCVD risk based on a representative group of U.S. adults.

The close correlation between dietary habit and ASCVD risk has been extensively proved. ASCVD risk would be increased by a Western Dietary (WD) pattern and decreased by Mediterranean Diet (MD) pattern and Dietary Approaches to Stop Hypertension (DASH) pattern (18, 19). Oxidative responses mediates the pathogenesis of ASCVD by its different influence on cellular damage, which would vary with aging (20). Such a damage can be relieved by dietary habit’s regulation on the redox status of human plasma. It has been shown that recommended, balanced dietary patterns such as MD and Atlantic Diet (AD) can provide all required macro- and micro-nutrients needed to maintain an organism in optimal balance and defend against oxidative damage (21, 22). Proper intake of antioxidants through food could keep our immune system in an optimal antioxidant state (23). In line with this, a recent study by He et al. suggested that high plasma antioxidant level might protect people against age-related diseases (24). Furthermore, growing research studies demonstrate that oxidative stress is closely related to cardiovascular disease and that total dietary antioxidant capacity (TDAC) is negatively associated with markers of inflammation such as C-reactive protein (CRP), platelet-activating factor (PAF), and adiponectin concentration (25–27). Further results from systematic review studies showed substantial associations between DTAC and most cardiovascular disease-related risk factors such as fasting glucose, blood pressure, CRP, and high-density lipoprotein cholesterol (HDL-C) (28). In addition, according to Detopoulou et al., PAF is implicated in atherosclerosis, and TDAC and healthy dietary patterns are inversely associated with PAF or its biosynthetic enzymes (29). Another investigator aiming to assess the relationship between adiponectin concentrations and TDAC in adults concluded that antioxidant foods benefit cardiovascular disease through an adiponectin-mediated pathway (27). This highlights the importance of implementing dietary modifications to increase the consumption of antioxidants to better prevent ASCVD risk.

We found that participants with 10-year risk of ASCVD ≥7.5% had lower dietary antioxidants scores in some commonly recognized elements that can alleviate oxidant responses, such as vitamin A, vitamin E, zinc, selenium, and carotenoids. Evidence suggested consumption above the recommended level of certain antioxidants can improve immune functioning and raise resistance to oxidative stress (12, 30). Dietary antioxidants, such as selenium, are beneficial in maintaining the optimal function of intracellular enzyme glutathione peroxide and extracellular protector selenoprotein P against oxidative stress in body (31). Moreover, previous studies have shown that carotenoids act as an essential precursor for the production of retinol such as vitamin A. Carotenoids themselves and their enzymatic products act as antioxidants in lipid-rich environment (32). Other antioxidants, such as vitamin C, can maximize neutrophil concentrations through dietary intake, reduce the production of reactive oxygen species during phagocytosis, and inhibit the oxidation of low-density lipoproteins (33, 34). Vitamin E is a group of fat-soluble compounds whose antioxidant activity mainly derives from α-tocopherol and γ-tocopherol. A lifestyle with Mediterranean diet score > 6 may provide better protection by affecting the oxidant/antioxidant balance in the body (21). Notably, no significant difference detected in vitamin C intake between two groups, which may attributes to the consensus that vitamin C-rich food such as fruits and vegetables may be beneficial to health (35). As the latest U.S. Dietary Guidelines for 2020–2025 emphasizes, there is a need to focus on the importance of healthy dietary patterns as a whole, rather than on individual nutrient, food, or food group in isolation (36).

Considering that these antioxidants might simultaneously affect ASCVD, we further analyzed whether overall potential of dietary antioxidant intake was associated with 10-year ASCVD risk. Present study revealed CDAI’s protective effect on 10-year ASCVD risk. As summarized by Senoner et al. (37), reactive oxygen species (ROS) negatively affect myocardial calcium handling and can promote atherosclerotic plaque formation, which was consistent with previous studies. Antioxidants may have a protective effect by modulating immune responses, viral replication, and gene expression to protect against ASCVD (30, 37, 38). Previous studies indicated that dietary total antioxidant capacity (TAC) may impact people with a cardiometabolic risk profile (39). Farhangi et al., indicated that dietary intake of zinc, selenium, and vitamins A, C, and E was inversely related to mortality risk (40). Similarly, Senoner and colleagues presented potential diets that might be beneficial in reducing the burden of oxidative stress in cardiovascular diseases (37). They take into account the difficulty of determining which specific components of food exert antioxidant effects, and therefore also recommend a diet consisting of a variety of foods containing different antioxidants, such as fresh fruits and vegetables and fish, rather than consuming a supplement consisting of a single antioxidant (37). In addition, they suggest that the benefits of antioxidants vary depending on the oxidative status of each individual (41).

After adjusting demographic and clinical covariates associated with ASCVD, the result of logistic regression still demonstrated a significant negative association between CDAI and 10-year ASCVD risk. However, this conclusion did not exclude the influence of other risk factors such as age, sex, hypertension, diabetes, and obesity, which may also be involved in the oxidation process of ASCVD. Age-related vascular endothelial dysfunction is a major antecedent to cardiovascular diseases. Brunt et al. conducted research that found supplementing trimethylamine-N-oxide (TMAO) in the diet increases circulating concentrations of trimethylamine-N-N-oxide, leading to greater oxidative stress caused by superoxides (42). This can result in reduced bioavailability of nitric oxide (NO) and endothelial dysfunction. As a result, healthy middle-aged and older adults have higher plasma TMAO levels compared to young control groups. In addition, accumulating evidence indicates that traditional risk factors for atherosclerosis, including hypertension and diabetes, would induce oxidative stress in blood vessels (43). Moreover, obesity increases the risk of atherogenic dyslipidemia and number of oxidative stress biomarkers. This might provide new ideas for early prevention and treatment of ASCVD. In contrast, some researchers have suggested an association between antioxidant-rich diets and atherosclerosis, but not with insulin, insulin resistance, or total cholesterol (44, 45). Similarly, a study by Kim et al. aimed at investigating the relationship between total antioxidant capacity in diets and supplements and cardiovascular disease risk factors in the NHANES found that intake of antioxidant-rich diets and supplements was beneficial in reducing the risk of cardiovascular disease, but that there were no significant associations between total antioxidant capacity of diets and blood pressure, total cholesterol, and blood glucose (46). The inconsistent results of the above studies regarding the relationship between antioxidant capacity and cardiovascular disease in different subgroups of the population suggest that there is also a strong need to isolate these covariates when exploring the relationship between CDAI and 10-year ASCVD risk. In addition, smoking is well known as a risk factor for cardiovascular disease, and results from a strong heart-based study exploring the potential moderating effects of several dietary nutrients with high antioxidant activity on cardiovascular disease in relation to exposure to environmental tobacco smoke showed that participants exposed to environmental tobacco smoke had a higher risk of cardiovascular disease compared with those not exposed, and a greater risk of cardiovascular disease compared with those who had higher vitamin E intake, as well as a greater risk of cardiovascular disease compared with those who had higher vitamin E intake, as well as a greater risk of cardiovascular disease compared with those who had higher vitamin E intake. The effects of environmental tobacco smoke on cardiovascular disease incidence were greater in those with low vitamin E intake compared to those with high vitamin E intake (47). Of note, the three study centers in the Strong Heart Study reported different smoking prevalence rates and used self-reported exposure to environmental tobacco smoke, making it difficult to differentiate between smoking, secondhand smoke, and thirdhand smoke, which limits the generalization of the results. Additional future studies are recommended to clarify whether smoking or environmental tobacco smoke exposure mediates the relationship between antioxidant capacity and 10-year ASCVD risk.

A stratified analysis was conducted to isolate the effect of CDAI from forementioned covariates. Interestingly, results of forest plot based-logistic regression and subgroup analysis of the generalized additive models revealed that the negative association between CDAI and 10-year ASCVD risk was robust to sex, age, hypertension, diabetes and BMI, proving the reliability and applicability of our results. This implies that the protective association we observed between dietary antioxidant intake and 10-year risk of ASCVD was independent of sex, age, hypertension, diabetes, and BMI status. Notably, recent medical developments suggest that antioxidants can neutralize free radicals and reduce the risk of disease caused by oxidative stress. However, in some cases, such as at high doses, antioxidants may act as pro-oxidants (48). Hence, the dose–response relationship between antioxidants and the 10-year risk of ASCVD needs further study. In addition, it is interesting to note that a previous study mentioned that there are many antioxidants, especially various safe plants that have antioxidant activity. They found that essential oils (EO) extracted from six chemical components acted as antioxidants and protected fish from oxidative stress (49). This implies that agents with antioxidant activity might be effective against ASCVD related to oxidative stress, and antioxidant drugs might be a new strategy for the prevention of ASCVD, and the relationship between the two and the mechanism of action still needs further study.

As far as we know, this is the initial research to examine the correlation between CDAI and 10-year ASCVD risk in a vast noninstitutionalized U.S. population based on NHANES data. Results suggested that CDAI was in significant negative association with 10-year ASCVD risk, providing clinical references to ASCVD prevention and control. The present study is mainly limited in three aspects. First, it could not construct or confirm any causal inference due to its cross-sectional design nature. Second, data used were all self-reported, which might be subjected to recall bias. Third, there are some potential covariates that are difficult to rule out, which may affect the relationship between CDAI and 10-year ASCVD risk.

## 5. Conclusion

Higher overall dietary antioxidant consumption was associated with lower 10-year ASCVD risk. The long-term impact of CDAI remains unclear and further analysis of data from longitudinal studies is needed to clarify the causal relationship between CDAI and 10-year ASCVD risk and its underlying mechanisms.

## Data availability statement

The raw data supporting the conclusions of this article will be made available by the authors, without undue reservation.

## Ethics statement

All data came from NHANES, which was approved by National Centre for Health Statistics Institutional Ethics Review Board, and all the subjects agreed on the survey and signed written consent. The patients/participants provided their written informed consent to participate in this study. The studies were conducted in accordance with the local legislation and institutional requirements.

## Author contributions

JZ: writing-most of manuscript, data curation, and processing. XL: writing-part of the manuscript and data curation. RW: writing-part of the manuscript. HN, LX, WW, CL, and JF: software, writing—review and editing, and supervision. YJ: methodology, writing—review and editing, and supervision. All authors contributed to the article and approved the submitted version.

## Funding

This study was supported by the Excellent Medical Youth Talent Project of the Shanghai Pudong New Area Health Commission (No. PWRq2020-34), the Xinglin Scholar Talent Program of Shanghai University of Traditional Chinese Medicine (No. 2022HLXL04), the Health Management Research Fund of the Shanghai Rehabilitation Medical Association (No. 2022KJCX047) and the Discipline Construction Plan of the Shanghai Pudong New Area Health Commission (No. PWZxk2022-06).

## Conflict of interest

The authors declare that the research was conducted in the absence of any commercial or financial relationships that could be construed as a potential conflict of interest.

## Publisher’s note

All claims expressed in this article are solely those of the authors and do not necessarily represent those of their affiliated organizations, or those of the publisher, the editors and the reviewers. Any product that may be evaluated in this article, or claim that may be made by its manufacturer, is not guaranteed or endorsed by the publisher.

## Footnotes

^1^http://www.empowerstats.com
